# Bortezomib inhibits chikungunya virus replication by interfering with viral protein synthesis

**DOI:** 10.1371/journal.pntd.0008336

**Published:** 2020-05-29

**Authors:** Parveen Kaur, Laura Sandra Lello, Age Utt, Sujit Krishna Dutta, Andres Merits, Justin Jang Hann Chu

**Affiliations:** 1 Laboratory of Molecular RNA Virology and Antiviral Strategies, Department of Microbiology and Immunology, Yong Loo Lin School of Medicine, National University of Singapore, Singapore; 2 Institute of Technology, University of Tartu, Tartu, Estonia; 3 School of Life Sciences and Chemical Technology, Ngee Ann Polytechnic, Singapore; 4 Collaborative and Translational Unit for HFMD, Institute of Molecular and Cell Biology, Agency for Science, Technology and Research (A*STAR), Singapore; Faculty of Science, Ain Shams University (ASU), EGYPT

## Abstract

Chikungunya virus (CHIKV) is an alphavirus that causes a febrile illness accompanied by myalgia and arthralgia. Despite having re-emerged as a significant public health threat, there are no approved therapeutics or prophylactics for CHIKV infection. In this study, we explored the anti-CHIKV effects of proteasome inhibitors and their potential mechanism of antiviral action. A panel of proteasome inhibitors with different functional groups reduced CHIKV infectious titers in a dose-dependent manner. Bortezomib, which has been FDA-approved for multiple myeloma and mantle cell lymphoma, was further investigated in downstream studies. The inhibitory activities of bortezomib were confirmed using different cellular models and CHIKV strains. Time-of-addition and time-of-removal studies suggested that bortezomib inhibited CHIKV at an early, post-entry stage of replication. In western blot analysis, bortezomib treatment resulted in a prominent decrease in structural protein levels as early as 6 hpi. Contrastingly, nsP4 levels showed strong elevations across all time-points. NsP2 and nsP3 levels showed a fluctuating trend, with some elevations between 12 to 20 hpi. Finally, qRT-PCR data revealed increased levels of both positive- and negative-sense CHIKV RNA at late stages of infection. It is likely that the reductions in structural protein levels is a major factor in the observed reductions in virus titer, with the alterations in non-structural protein ratios potentially being a contributing factor. Proteasome inhibitors like bortezomib likely disrupt CHIKV replication through a variety of complex mechanisms and may display a potential for use as therapeutics against CHIKV infection. They also represent valuable tools for studies of CHIKV molecular biology and virus-host interactions.

## Author summary

Chikungunya virus (CHIKV) is a mosquito-transmitted virus that causes an illness with debilitating muscle and joint pain. CHIKV has infected millions in a continued wave of outbreaks worldwide. Despite this, there are no approved antivirals or vaccines against CHIKV infection. In this study, we explored the inhibitory effects of proteasome inhibitors against CHIKV. A panel of proteasome inhibitors was found to reduce CHIKV titres in CHIKV-infected cells. We selected bortezomib, an FDA-approved drug, for further investigation into its antiviral mechanism. We confirmed the anti-CHIKV effects of bortezomib using different cell lines and CHIKV strains. We found that bortezomib resulted in a major decrease in levels of CHIKV structural proteins, which are involved in formation of progeny virus particles. Bortezomib treatment also prominently increased synthesis of viral replicase components and increased CHIKV RNA synthesis. We propose that proteasome inhibitors like bortezomib are likely to inhibit CHIKV through various mechanisms that ultimately lead to a decrease in structural proteins and infectious viral progeny. This study suggests that proteasome inhibitors display a potential for further development as antivirals against CHIKV infection and may be useful tools to study CHIKV molecular biology and virus-host interactions.

## Introduction

Chikungunya virus (CHIKV) is a mosquito-borne virus that has re-emerged as a major public health threat in the last decade [1, 2]. CHIKV infection results in a febrile illness accompanied by debilitating polyarthralgia, myalgia and maculopapular rash [3, 4]. Chronic polyarthralgia lasting for several months to years has been reported in a subset of patients, significantly compromising quality of life [3, 5, 6]. While historically confined to Asia and sub-Saharan Africa, CHIKV outbreaks have recently also been reported in non-endemic areas, including islands in the Indian and Pacific Oceans, parts of Europe, as well as countries in the Americas, infecting millions [2, 7–9]. Factors contributing to the continued waves of CHIKV epidemics worldwide include increased global travel and rising global temperatures, which have resulted in wider distribution of the mosquito vectors, *Aedes aegypti* and *Aedes albopictus* [8, 10, 11]. Despite the significant medical threat posed by CHIKV, there are currently no licensed therapeutics or prophylactics against CHIKV infection. There remains an urgent need for the discovery of novel antivirals against CHIKV infection, accompanied by an improved understanding of CHIKV replication and pathogenesis.

CHIKV belongs to the genus *Alphavirus* in the *Togaviridae* family [12]. CHIKV is part of the Old World alphaviruses, which also include the well-studied model viruses, Semliki Forest virus (SFV) and Sindbis virus (SINV) [13]. Chikungunya virions are enveloped, with a positive-sense RNA genome enclosed within a nucleocapsid core [12]. The CHIKV genome is approximately 11.8 kb long and contains two open reading frames (ORF): a 7.4 kb ORF encoding the non-structural (ns) proteins (nsP1, nsP2, nsP3 and nsP4), and a 3.7 kb ORF encoding the structural proteins (capsid, E3, E2, 6K/TF and E1) [12, 14]. Glycoprotein spikes consisting of E1 and E2 on the CHIKV envelope mediate virion binding and entry into host cells by receptor-mediated endocytosis [15, 16]. Within the host cell, the viral genome is translated by the eukaryotic translation machinery, producing the polyprotein precursor for the ns proteins, which is then processed into mature proteins. The ns proteins complex with viral genome and cellular components to form the viral replicase [17]. The viral replicase generates the full-length negative-sense CHIKV RNA, which serves as a template for the transcription of full-length positive-sense genomic (G), as well as the subgenomic (SG) CHIKV RNAs [18]. This is followed by translation of the viral structural proteins from the SG RNA [13]. In the later stages of the CHIKV replication cycle, the nucleocapsid core assembles in the cytoplasm and buds out through the plasma membrane, acquiring a lipid envelope to form mature, infectious progeny [19, 20].

The ubiquitin-proteasome system is the major pathway for degradation of cellular proteins that are damaged, misfolded, or require downregulation [21]. Proteins targeted for proteasomal degradation are usually covalently tagged with at least four lysine-48 (K48)-linked ubiquitin subunits [21, 22]. Accumulating evidence suggests that the ubiquitin-proteasome system is involved in the replication of several positive-sense RNA viruses [23]. The ubiquitin-proteasome system has been found to control intracellular levels of certain viral proteins during replication, including viral proteases, structural proteins and RNA-dependent RNA polymerase (RdRp) [24]. For instance, the proteasomal degradation of viral proteases has been well documented for picornaviruses, including encephalomyocarditis virus and hepatitis A virus, [25–27]. Given the role of the viral protease in triggering apoptosis, researchers speculate that the UPS may be utilized by picornaviruses to maintain sufficiently low levels of the viral protease during early replication to prevent premature apoptosis [28, 29]. Early studies on SINV and SFV suggested that nsP4, which functions as the RdRp, is degraded by the proteasome due to the presence of a highly conserved destabilizing tyrosine residue at the N-terminus [30–32]. In a study with SINV mutants, Shirako and colleagues (1998) found that only nsP4 proteins with N-terminal Tyr, His, Phe or Trp have wild-type activity for RNA replication [30, 31]. The authors suggested that the metabolic instability of nsP4 is not necessary for its function. Instead, they suggested that SINV nsP4 requires an aromatic amino acid or histidine at its N-terminus, and its metabolic instability is simply a consequence of this requirement [31]. Proteasomal inhibition has been found to reduce titers of several alphaviruses, including SFV, Mayaro virus and Una virus [32, 33]. For studies on CHIKV, proteomic analyses have revealed differential regulation of several proteasome subunits upon infection, although results differed between studies [34, 35]. For instance, upon CHIKV infection, PSMA6 (proteasome subunit alpha type-6) was reported to be downregulated in one study [34] and upregulated in another [35]. In addition, treatment with the proteasome inhibitors MG132 and lactacystin resulted in a reduction of viral titers in CHIKV-infected GripTite 293 MSR cells [36].

In this study, we explored the antiviral potential of a panel of proteasome inhibitors. Bortezomib, a proteasome inhibitor approved for the treatment of multiple myeloma and mantle cell lymphoma [37, 38], was selected for downstream experiments to investigate the potential mechanism of antiviral action. Bortezomib treatment was found to interfere with a post-entry step in the CHIKV replication cycle. Treatment with bortezomib also resulted in a decrease in structural protein expression. Western blot analysis showed elevated levels of CHIKV nsP4 from early time-points, suggesting the possibility that the CHIKV nsP4 is degraded by the proteasome, similar to the nsP4 of SFV and SINV. In addition, a strong reduction in structural protein levels was also observed. Levels of both positive-sense and negative-sense CHIKV RNA were elevated at later time-points post-infection. This study shows that proteasome inhibitors affect different processes involved in CHIKV replication and may serve as potential antivirals against CHIKV. Further work into their clinical therapeutic use during CHIKV infection is warranted.

## Results

### Proteasome inhibitors suppress CHIKV infection

**Fig 1:**
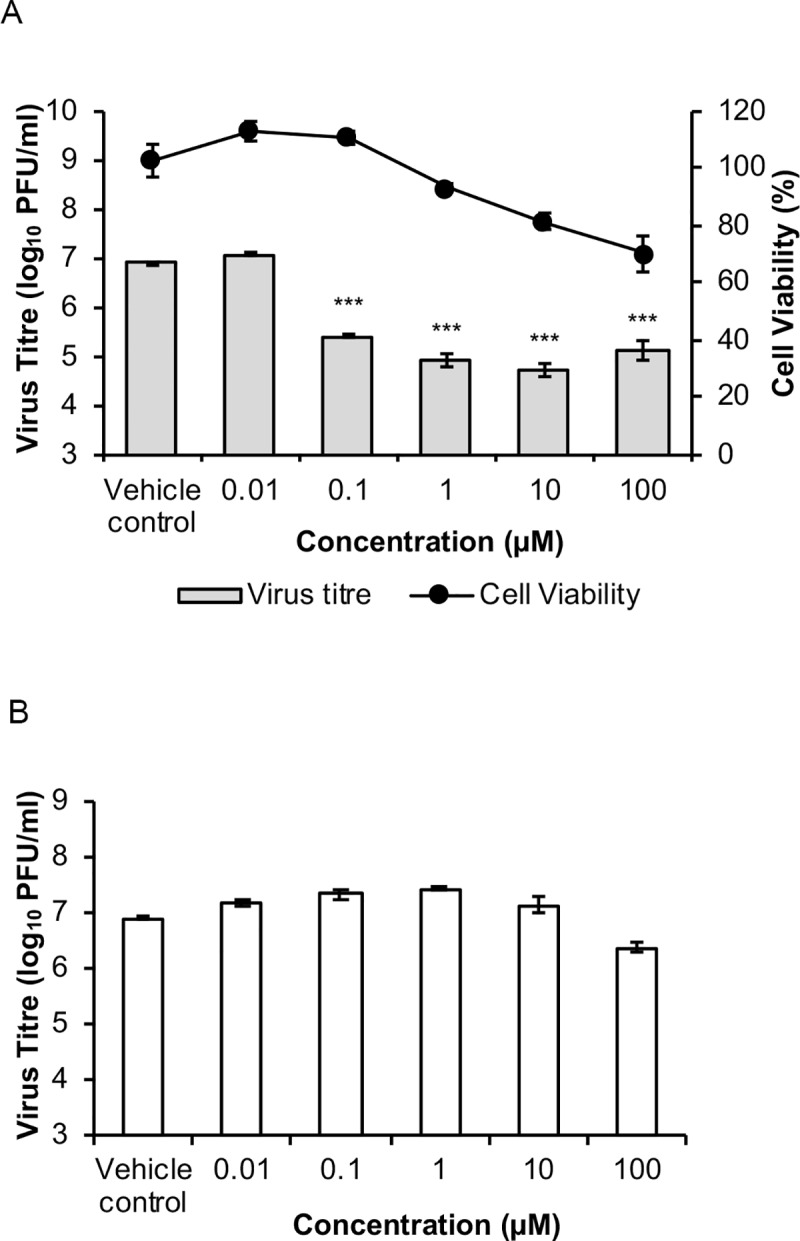
Effects of bortezomib treatment on CHIKV infection in BHK21 cells. (A) BHK21 cells were infected with CHIKV-122508 and treated with indicated concentrations of bortezomib for 24h. CHIKV titers are presented in the bar graphs corresponding to the primary axis. Cell viability is presented by the line graph corresponding to the secondary axis. Error bars represent standard errors of means from two independent experiments done in triplicates. *** *P* ≤ 0.001 (one-way ANOVA with Dunnett’s post-test comparing bortezomib-treated samples to vehicle control). (B) In pre-treatment assays, BHK21 cells were treated with bortezomib for 2h, washed and infected with CHIKV-122508 at MOI 10. Supernatants were harvested at 24h for plaque assays. Error bars represent standard errors of means from three technical replicates.

In order to explore the anti-CHIKV effects of proteasome inhibitors, we tested the inhibitory potential of bortezomib using CHIKV-infected BHK21 cells in both post-treatment and pre-treatment assays. For post-treatment assays, BHK21 cells were infected with CHIKV-122508 prior to treatment with bortezomib. This was performed to establish the effects of bortezomib on post-entry steps. As seen in Fig 1A, treatment with 0.1 μM bortezomib onwards resulted in significant inhibition of CHIKV infectious titer. A maximal 98.5% (2.2 log_10_ units) inhibition was obtained with 10 μM treatment of bortezomib. Cell viability remained above 70% across all concentrations (Fig 1A), indicating minimal toxicity. In fact, treatment with 0.1 μM bortezomib produced 97% (1.5 log_10_ units) inhibition with no decrease in cell viability, suggesting that bortezomib-mediated reduction in viral titers were not due to cytotoxicity. A pre-treatment assay was also carried out, where BHK21 cells were treated with bortezomib for 2 hours, washed with PBS and then immediately infected with CHIKV. This was to determine whether bortezomib could affect the cells in a way that hindered CHIKV attachment or entry. The inhibitory trend was not observed upon pre-treatment with bortezomib (Fig 1B). Collectively, this data suggests that the anti-CHIKV effects of bortezomib are likely to occur at a post-entry stage in the replication cycle.

**Fig 2:**
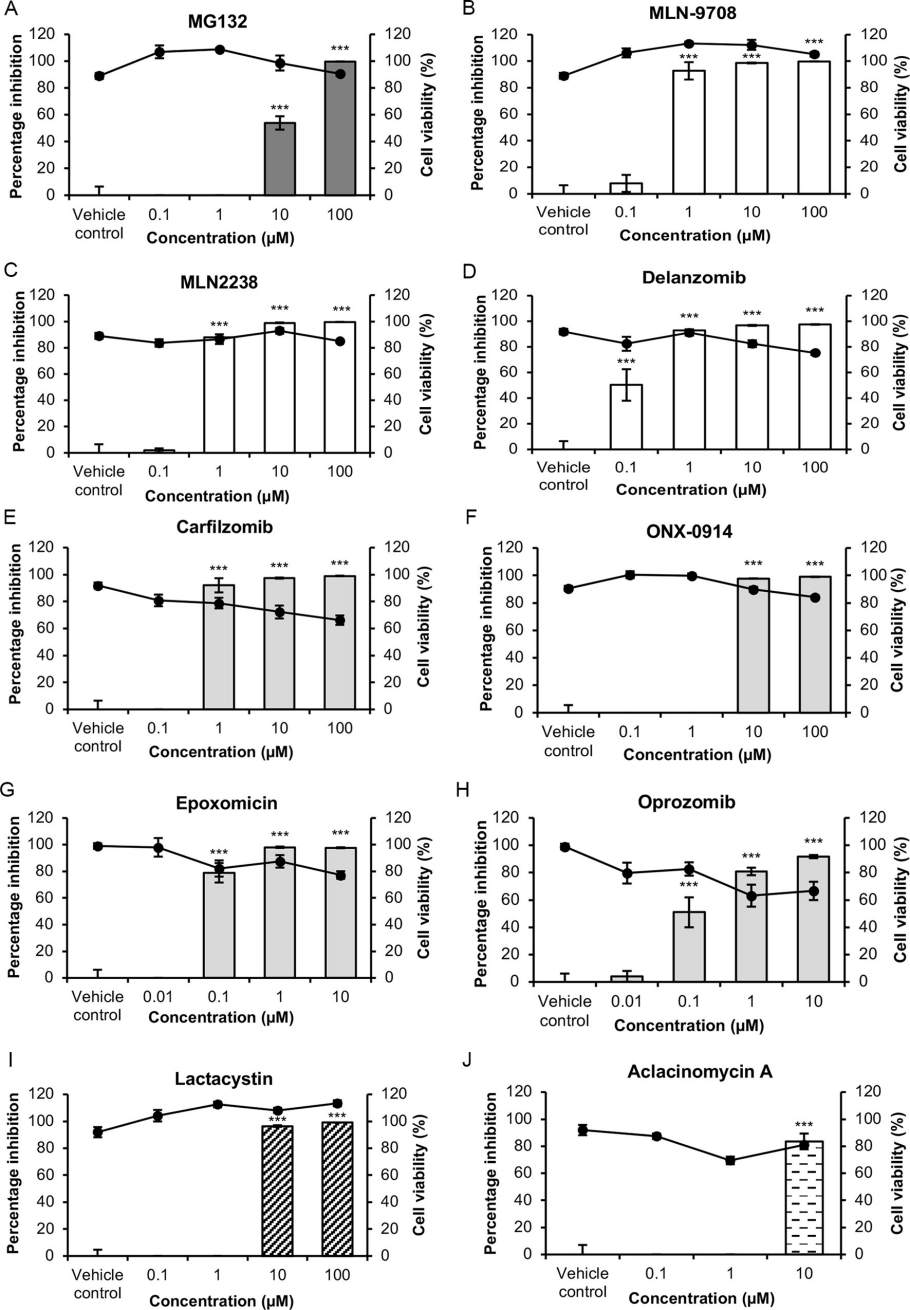
Effects of proteasome inhibitors on CHIKV infection in BHK21 cells. BHK21 cells were infected with CHIKV-122508 and treated with indicated concentrations of (A) MG-132, (B) MLN-9708 (C) MLN-2238, (D) delanzomib, (E) carfilzomib (F) ONX-0914, (G) epoxomicin, (H) oprozomib (I) lactacystin and (J) aclacinomycin A. Virus supernatants were harvested for plaque assays at 24 hpi. Percentage inhibition of CHIKV titers by proteasome inhibitors are presented in the bar graphs corresponding to the primary axis. Percentage inhibition was calculated after normalisation to vehicle controls. Cell viabilities are presented by the line graph corresponding to the secondary axis. Error bars represent standard errors of means from three technical replicates. Proteasome inhibitors tested belong to different compound classes, namely peptide aldehydes: MG132; peptide boronates: MLN-9708, MLN-2238 and delanzomib; peptide epoxyketones: carfilzomib, ONX-0914, epoxomicin and oprozomib; β-lactones: lactacystin; anthracycline derivatives: aclacinomycin A. *** *P* ≤ 0.001 (one-way ANOVA with Dunnett’s post-test comparing proteasome inhibitor-treated samples to vehicle control).

In order to confirm the antiviral effects of proteasome inhibition on CHIKV infection, a panel of proteasome inhibitors from compound classes with different functional groups (S1 Table) was screened in post-treatment assays in BHK21 cells. As shown in Fig 2, most proteasome inhibitors inhibited CHIKV replication at concentrations that were minimally cytotoxic, indicating that proteasomal inhibition is detrimental to CHIKV infection. The inhibition in CHIKV titers observed for MG132 (Fig 2A) and lactacystin (Fig 2I) also supports previous data by Karpe and colleagues [36]. Inhibitors which exhibited at least a 97% maximal inhibition came from various compound classes and included MG-132 (Fig 2A), MLN-9708 (Fig 2B), MLN-2238 (Fig 2C), delanzomib (Fig 2D), ONX-0914 (Fig 2F), epoxomicin (Fig 2G) and lactacystin (Fig 2I). Aclacinomycin A (Fig 2J) treatment resulted in an 84% inhibition at 10 μM concentration. Treatment with 100 μM of aclacinomycin A resulted in 100% cell death, likely due to its inhibitory effects on macromolecular synthesis [39]. Treatment with carfilzomib (Fig 2E) and oprozomib (Fig 2H) resulted in decreasing cell viabilities, suggesting that the inhibition in CHIKV titers observed for these two compounds may be a result of cytotoxicity. In summary, results from Fig 2 suggested that the proteasome may play a pro-viral role in CHIKV replication. It is likely that the varying mechanisms of inhibition by different proteasomal inhibitors influenced the magnitude of inhibition of CHIKV infection.

### Inhibitory effects of bortezomib across different CHIKV strains and cell lines

**Fig 3:**
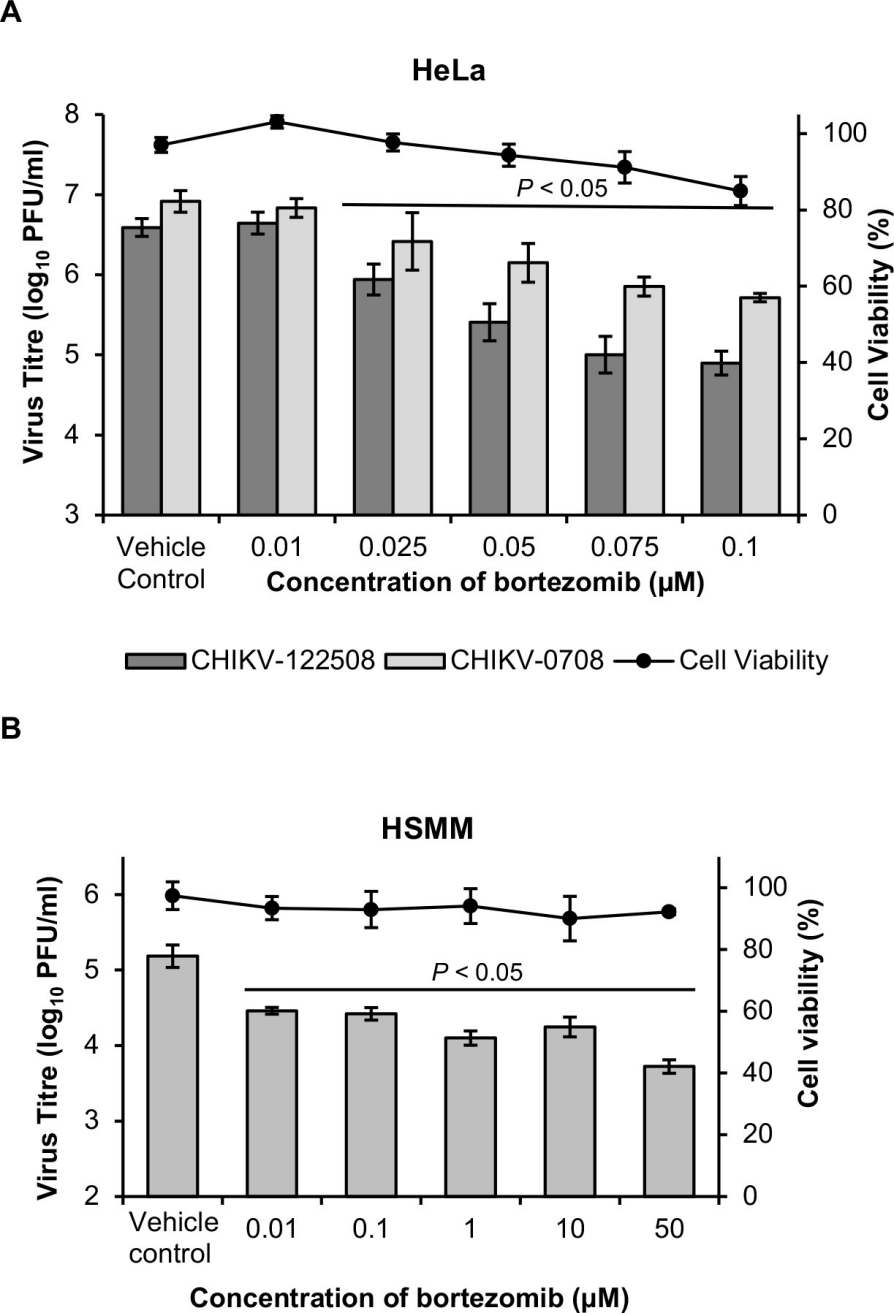
Inhibitory effects of bortezomib on different CHIKV strains and cell lines. (A) HeLa cells were infected with CHIKV-122508 or CHIKV-0708 and post-treated with bortezomib for 24h. CHIKV titers are presented in the bar graphs corresponding to the primary axis. Cell viability is presented by the line graph corresponding to the secondary axis. Error bars for CHIKV-122508 represent standard errors of means from five independent experiments done in triplicates. Error bars for CHIKV-0708 represent standard errors of means from two independent experiments done in triplicates. (B) HSMM cells were infected with CHIKV-122508 and post-treated with bortezomib for 24h. CHIKV titers are presented in the bar graphs corresponding to the primary axis. Cell viability is presented by the line graph corresponding to the secondary axis. Error bars represent standard errors of means from at least three technical replicates. Statistical significance of treated samples compared to vehicle control was analysed using one-way ANOVA test and Dunnett’s post-test.

In order to confirm that the inhibitory effects observed were not due to cell-line- or CHIKV strain-dependent effects, bortezomib was tested in two additional cell lines (HeLa and HSMM) and against two strains of CHIKV. In HeLa cells, bortezomib treatment resulted in a dose-dependent reduction of titer for both CHIKV strains (Fig 3A). A maximal inhibition of 1.8 log_10_ units and 1.2 log_10_ PFU/ml was observed with CHIKV-122508 and CHIKV-0708, respectively (Fig 3A). The efficacy of bortezomib was also confirmed in primary human skeletal myoblasts (HSMM), an *in vivo* target of CHIKV infection [40, 41]. In agreement with results from BHK21 and HeLa cells, CHIKV replication in HSMM cells was inhibited in a dose-dependent manner upon bortezomib treatment (Fig 3B). A maximal inhibition of 1.5 log_10_ units was observed in CHIKV-infected HSMM cells upon treatment with 50 μM bortezomib. For investigation into the mechanism of antiviral action of bortezomib, downstream studies were conducted in HeLa cells infected with CHIKV-122508 at MOI 10 (SI = 20.6, Table 1). HeLa cells were chosen for downstream studies as it is a human cell line in which high CHIKV titers of >10^6^ PFU/ml were obtained.

**Table 1:** Selectivity indices of bortezomib treatment in BHK, HeLa and HSMM cells infected with CHIKV-122508 at MOI 10.

	BHK	HeLa	HSMM
CC_50_ (μM)	>100	0.4737	>100
EC_50_ (μM)	0.0763	0.023	<0.01
SI (CC_50_ /EC_50_)	**>1310**	**20.6**	**>10,000**

### Time-of-addition and time-of-removal studies in HeLa cells

**Fig 4:**
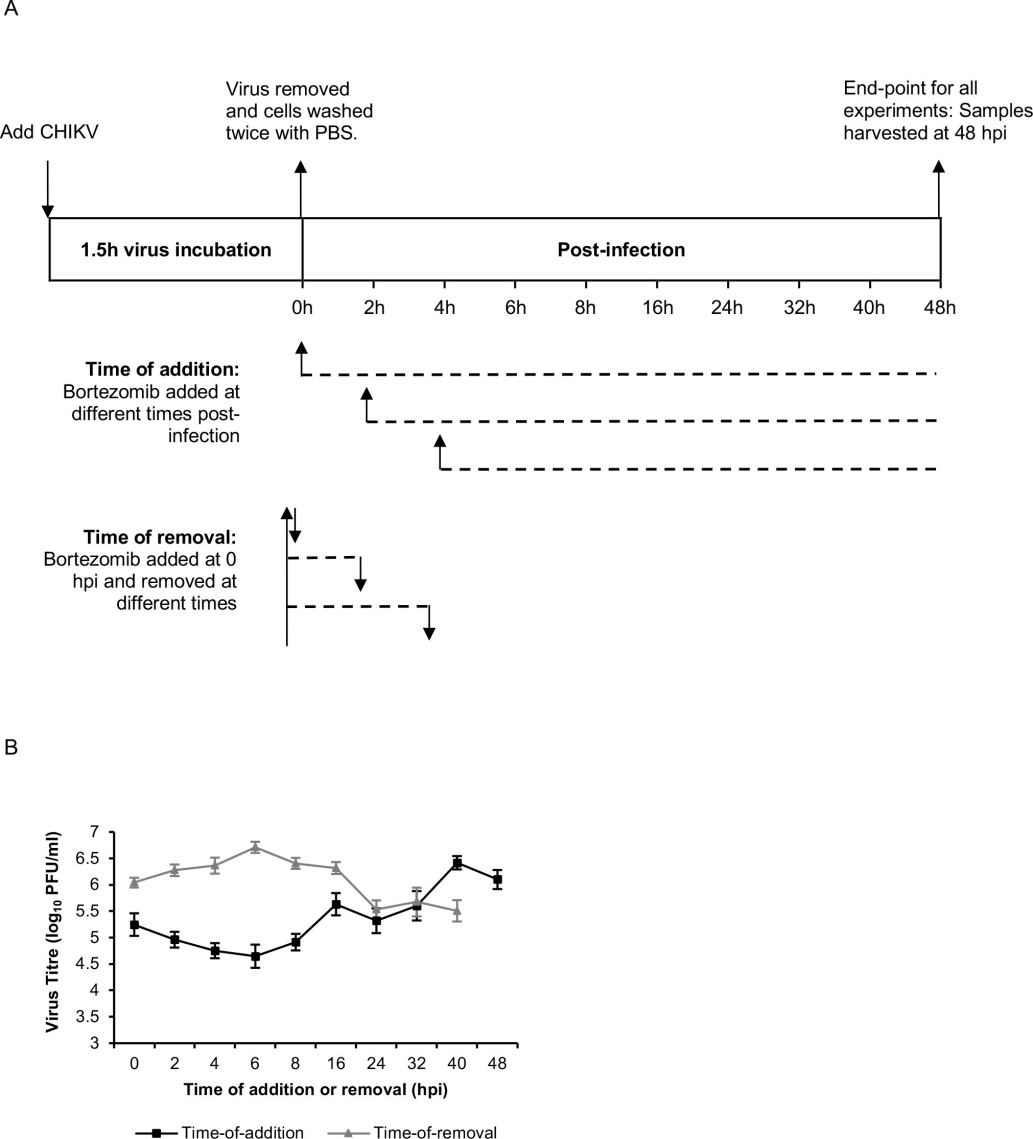
Time-of-addition and time-of-removal studies. HeLa monolayers were first infected with CHIKV-122508 for 1.5h. (A) The schematic diagram for the first three time-points (0, 2, 4 hpi) are shown. For time-of-addition studies, bortezomib was added at different time-points post-infection. For time-of-removal studies, bortezomib was added at 0h post-infection and then removed at different times. All samples were harvested at 48 hpi. Dotted lines represent the duration of bortezomib treatment. (B) CHIKV titers from time-of-addition and time-of-removal assays. Error bars represent standard error of means from at least 4 replicates.

As a preliminary step towards elucidating the mechanism of action of the bortezomib, time-of-addition and time-of-removal studies were performed in HeLa cells to identify the window in the CHIKV replication cycle where bortezomib exerts its effects. As displayed in the schematic diagram in Fig 4A, time-of-addition experiments were performed by adding bortezomib at different time-points post-infection, while time-of-removal experiments were conducted by adding bortezomib immediately after infection and removing it at different time-points post-infection.

The antiviral effect of bortezomib was most pronounced when it was added at early time-points (0-8h) after infection, with a reduced effect between 16–32 hours post-infection (hpi), indicating that a post-entry step in the replication cycle was inhibited (Fig 4B). The time-of-removal experiment revealed that removal of bortezomib at 16 hpi or earlier prevented its inhibitory effects (Fig 4B). Furthermore, time-of-removal studies also showed that bortezomib treatment for the first 16h was necessary before its inhibitory effect could be observed.

### Bortezomib treatment causes a decrease in structural protein levels

**Fig 5:**
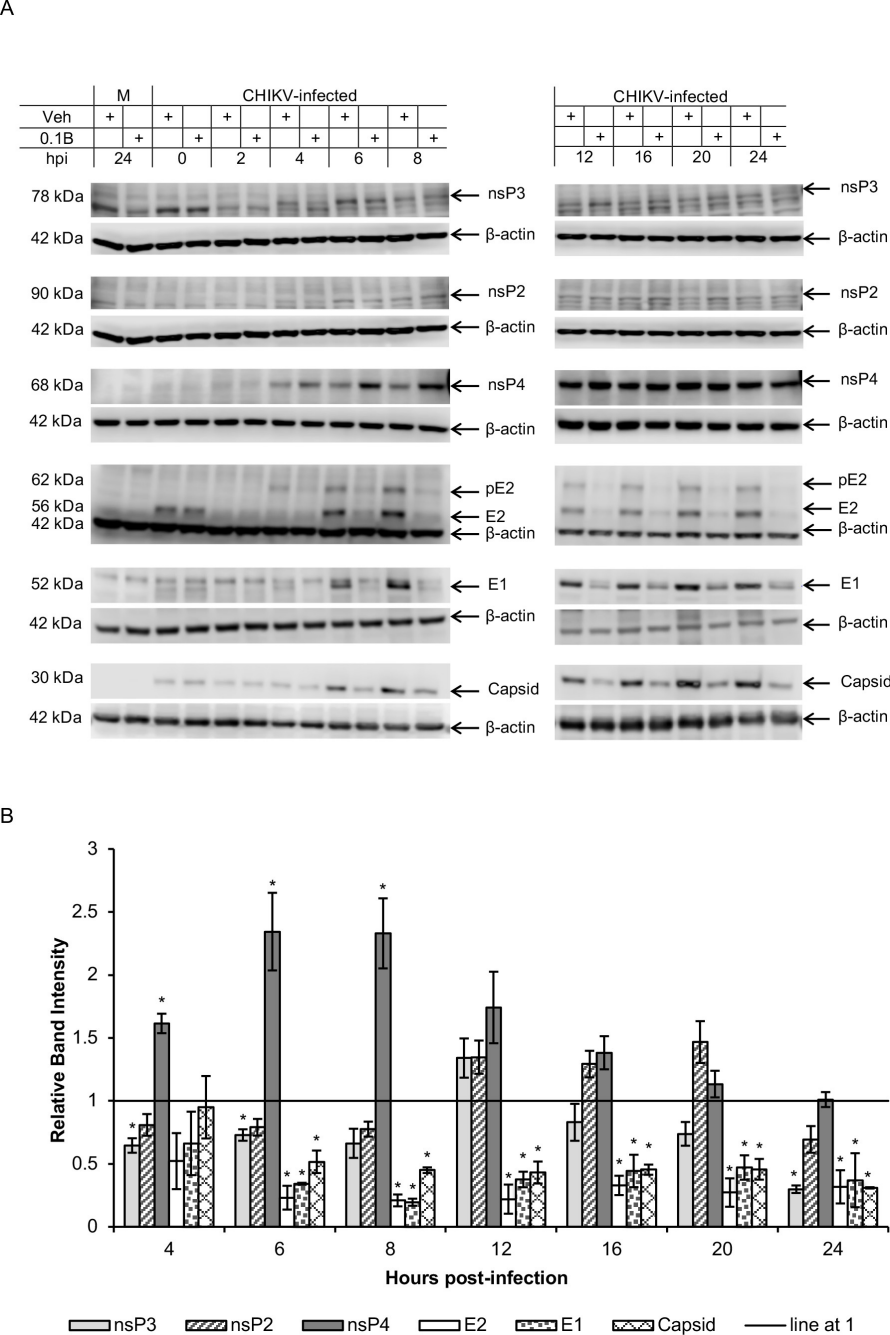
Time-point studies of CHIKV protein levels upon bortezomib treatment. HeLa cells were infected with CHIKV-122508 and treated with either 0.1 μM bortezomib or vehicle control. Protein samples were harvested at different time-points post-infection and probed for CHIKV proteins. (A) Western blot images are representative of two independent experiments. Actin was added as a loading control. Actin for nsP3 and nsP2 are the same because both proteins were probed from the same blot. M: Mock, Veh: Vehicle control, 0.1B: 0.1 μM bortezomib, hpi: hours post-infection (refers to time-points when samples were harvested), +: indicates treatment with either Veh or 0.1B. (B) Band intensities of bortezomib-treated samples relative to vehicle controls for each time-point are shown in the bar graphs. Band intensities were calculated after normalisation with actin for each sample. Error bars represent standard error of means from two independent experiments. * *P* < 0.05 (Welch’s t-test comparing proteasome inhibitor-treated samples to vehicle control at each time-point for each CHIKV protein).

**Fig 6:**
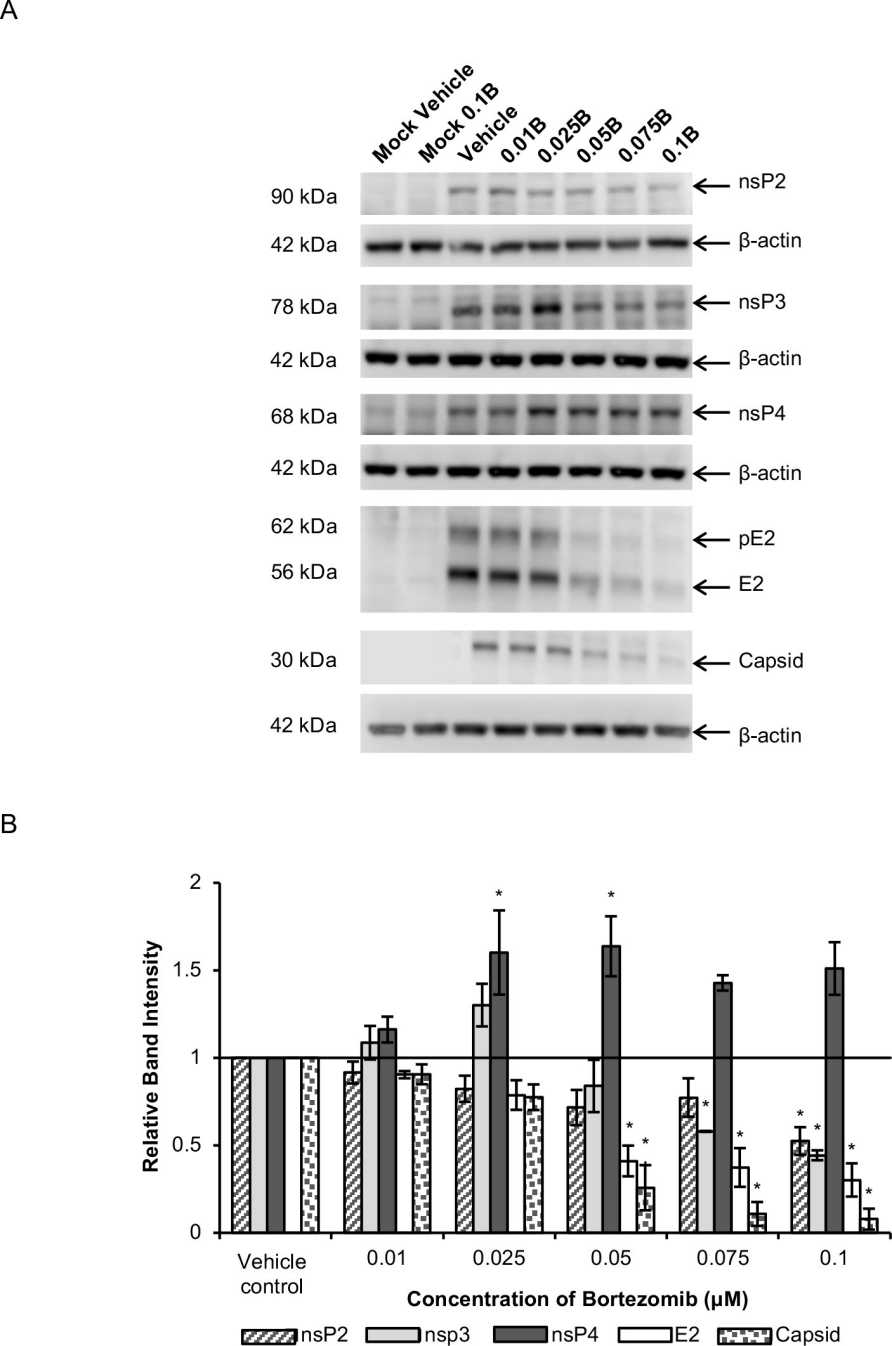
Dose-dependent studies of CHIKV protein levels upon bortezomib treatment at 6 hpi. HeLa cells were infected with CHIKV-122508 and treated with either 0.1 μM bortezomib or vehicle control. Protein samples were harvested at 6 hpi and probed for CHIKV proteins. (A) Western blot images are representative of three independent experiments. Actin was added as a loading control. E2 and capsid were probed on the same blot and share the same actin bands. 0.01B: 0.01 μM bortezomib, 0.025B: 0.025 μM bortezomib, 0.05B: 0.05 μM bortezomib, 0.075B: 0.075 μM bortezomib, 0.1B: 0.1 μM bortezomib. (B) Band intensities of bortezomib-treated samples relative to vehicle control are presented in the bar graph. Band intensities were calculated after normalisation with actin for each sample. Error bars represent standard error of means from three independent experiments. * *P* < 0.05 (one-way ANOVA with Dunnett’s post-test comparing bortezomib-treated samples to vehicle control for each CHIKV protein).

We next evaluated the possibility that bortezomib affects CHIKV RNA synthesis and/or viral RNA translation events. To investigate this further, the levels of various CHIKV proteins were measured at different time-points post-infection in HeLa cells. As shown in Fig 5, bortezomib treatment resulted in a striking decrease of about 50 to 80% in levels of the CHIKV structural proteins, E2, E1 and capsid, from 6 hpi onwards. Correspondingly, structural protein levels were also observed to decrease in a dose-dependent manner upon treatment with different concentrations of bortezomib at 6 hpi (Fig 6).

Unlike the consistent and significant reductions observed for structural proteins, the levels of nsP2 and nsP3 fluctuated across different time-points (Fig 5). There were minor increases in nsP3 levels at 12 hpi (35% increase). At all other time-points, nsP3 levels in bortezomib-treated samples were about 20% to 35% lower than vehicle-treated samples, except for 24 hpi, where it was 70% lower. An increase in nsP2 levels was also observed from 12 hpi (35% increase) to 20 hpi (47% increase). At all other time-points, levels of nsP2 was about 20% to 30% lower than in untreated cells. At 6 hpi, a dose-dependent decrease in the levels of nsP2 and nsP3 was observed upon treatment with increasing concentrations of bortezomib (Fig 6). These fluctuations suggest that bortezomib may affect structural and ns proteins in different ways, and there may be multiple competing effects on ns proteins, leading to the observed results. Bortezomib may cause an increase in nsP2/nsP3 expression or reduction in degradation, which may be counteracted by the reduced spreading of CHIKV in the monolayer as a result of diminished virion production during each infection cycle.

Western blot analysis showed major elevations in nsP4 levels upon bortezomib treatment, especially at early time-points of 6 and 8 hpi (Fig 5). The band intensities of nsP4 in bortezomib-treated samples were about 60% to 130% higher than vehicle controls from 4 hpi to 20 hpi (Fig 5B). The levels of nsP4 for bortezomib-treated samples were elevated at concentrations from 0.025 μM to 0.1 μM (Fig 6), which are the same treatment concentrations that resulted in reduction of infectious CHIKV titers for CHIKV-122508 in HeLa cells (Fig 3A). However, this increase in nsP4 levels was not dose-dependent (Fig 6), suggesting that the elevated nsP4 levels may not be the main pathway contributing to the anti-CHIKV effects of bortezomib. The increase in nsP4 levels upon proteasome inhibition may suggest that the CHIKV nsP4 is degraded by the proteasome, similar to the nsP4 of SFV and SINV [30, 31, 42]. Indeed, the CHIKV nsP4 also contains a conserved destabilizing N-terminal Tyr residue [12]. To identify potential ubiquitination sites in CHIKV proteins, we ran the protein sequences of all CHIKV proteins in UbPred, a random forest-based predictor of protein ubiquitination sites [43]. The CHIKV nsP4 protein showed the highest number of predicted ubiquitination sites (S2 Table) with confidence levels of medium or high, further supporting the possibility that the CHIKV nsp4 is a substrate for the proteasome.

It is interesting to note that, for both structural and ns proteins, differences between bortezomib-treated samples and vehicle controls were apparent from 4 to 6 hpi onwards in western blot data. This is congruent with the time-of-addition studies (Fig 4B), which showed that the maximal inhibitory effect of bortezomib was observed when added between 0 to 8 hpi.

**Fig 7:**
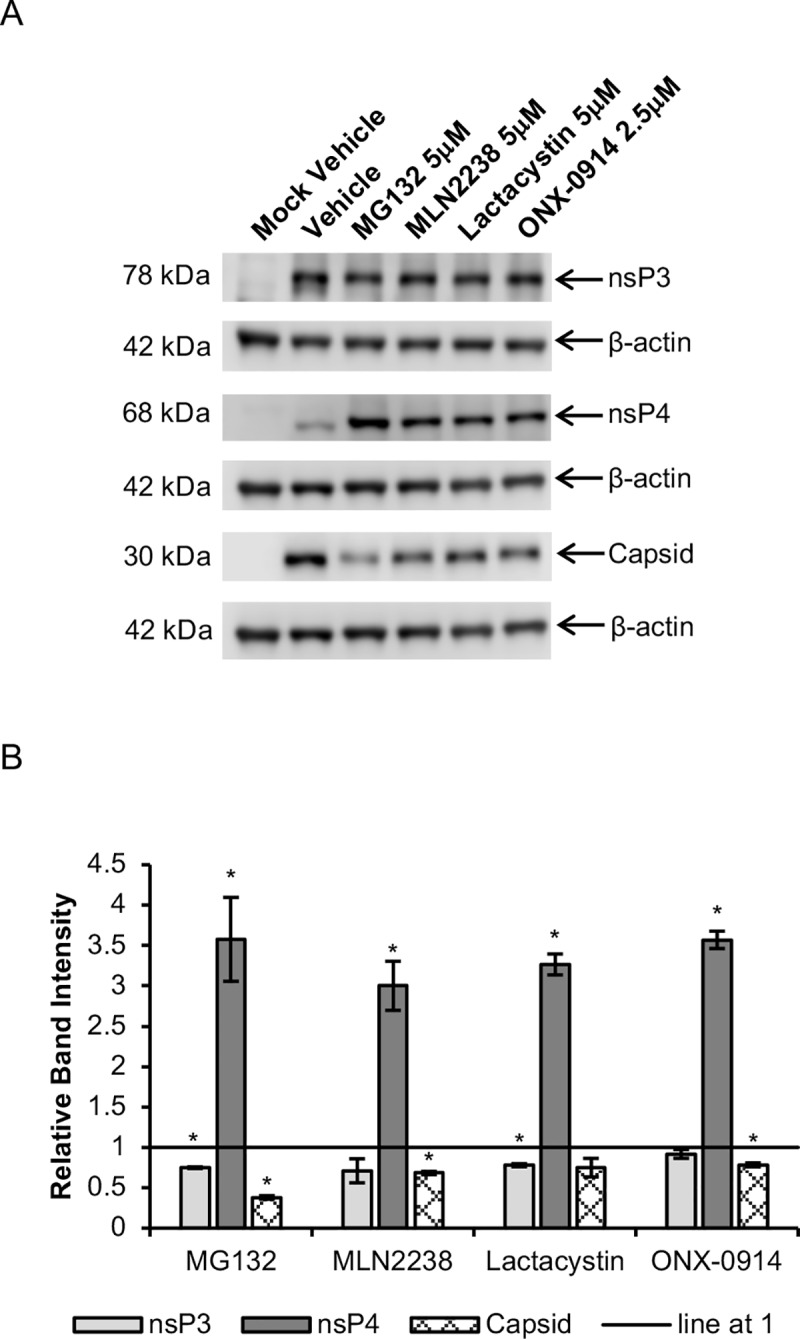
Changes in CHIKV protein levels upon treatment with various proteasome inhibitors at 6 hpi. HeLa cells were infected with CHIKV-122508 and treated with various proteasome inhibitors at the indicated concentrations or vehicle control. Protein samples were harvested at 6 hpi and probed for CHIKV proteins. (A) Western blot images are representative of two independent experiments. Actin was added as a loading control. NsP4 and capsid were probed on the same blot and share the same actin bands. (B) Band intensities of proteasome inhibitor-treated samples relative to vehicle control are presented in the bar graph. Band intensities were calculated after normalisation with actin for each sample. Error bars represent standard error of means from two independent experiments. * *P* < 0.05 (Welch’s t-test comparing proteasome inhibitor-treated samples to vehicle control for each CHIKV protein).

To determine whether the bortezomib-induced changes in CHIKV protein levels extended to other proteasome inhibitors as well, we treated CHIKV-infected cells with a number of other proteasome inhibitors carrying different functional groups. Similar to bortezomib, treatment with MG132, MLN2238, lactacystin or ONX-0914 resulted in a reduction in band intensities of capsid (22% to 62% decrease) and nsP3 (9% to 29% decrease) at 6 hpi (Fig 7). Likewise, band intensities of nsP4 were 200% to 260% greater in inhibitor-treated samples when compared against vehicle controls (Fig 7). This suggests that anti-CHIKV mechanisms of proteasome inhibitors are likely to involve the same pathways, despite differences in functional groups.

Alphavirus infections are known to induce translational shut-off through a variety of mechanisms, including phosphorylation of eIF2α by PKR [44]. Even though phosphorylated eIF2α inhibits global protein synthesis, translation of alphavirus SG RNA remains unaffected [45]. In fact, in infected cells, SG RNA translation occurs more efficiently in the presence of eIF2α phosphorylation [46]. However, during early alphavirus infection, eIF2α phosphorylation appears to be inhibited by nsP4, possibly to allow accumulation of CHIKV RNA transcripts for subsequent translation [47].

**Fig 8:**
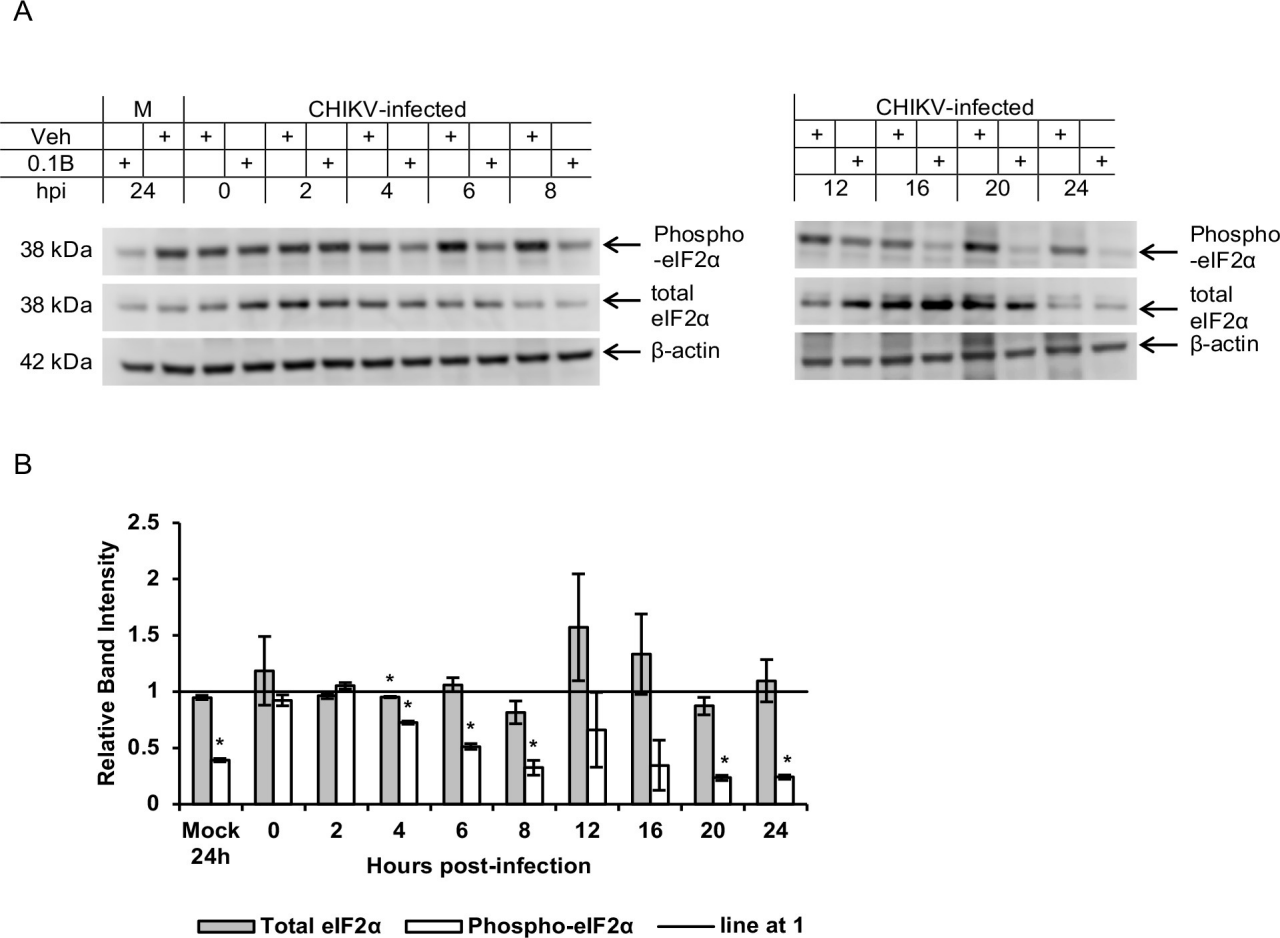
Changes in total and phospho-eIF2α levels upon bortezomib treatment. (A) HeLa cells were infected with CHIKV-122508 and treated with either 0.1 μM bortezomib or vehicle control. Protein samples were harvested at different time-points post-infection and probed for phospho-eIF2α and total eIF2α. Western blot images are representative of two independent experiments. Actin was added as a loading control. M: Mock, Veh: Vehicle control, 0.1B: 0.1 μM bortezomib, hpi: hours post-infection (refers to time-points when samples were harvested), +: indicates treatment with either Veh or 0.1B. (B) Band intensities of bortezomib-treated samples relative to vehicle controls for each time-point are shown in the bar graph. Band intensities were calculated after normalisation with actin for each sample. Error bars represent standard error of means from two independent experiments. * *P* < 0.05 (Welch’s t-test comparing proteasome inhibitor-treated samples to vehicle control for each CHIKV protein).

To understand the effects of bortezomib treatment on eIF2α phosphorylation in CHIKV-infected cells, the levels of phosphorylated (Ser51) eIF2α as well as total eIF2α were measured in time-point studies. Fig 8 shows that bortezomib treatment of CHIKV-infected HeLa cells caused a general reduction in levels of phospho-eIF2α over time, with relatively stable levels of total eIF2α. Strikingly, however, bortezomib treatment in mock-infected cells also resulted in reduced phospho-eIF2α levels (approximately 60% reduction) at 24h post-treatment. This indicated that the decrease in phospho-eIF2α levels may not be due to viral ns proteins (including nsP4) accumulation; rather, an alternative pathway that was activated by bortezomib.

### Bortezomib treatment increases in CHIKV RNA levels

**Fig 9:**
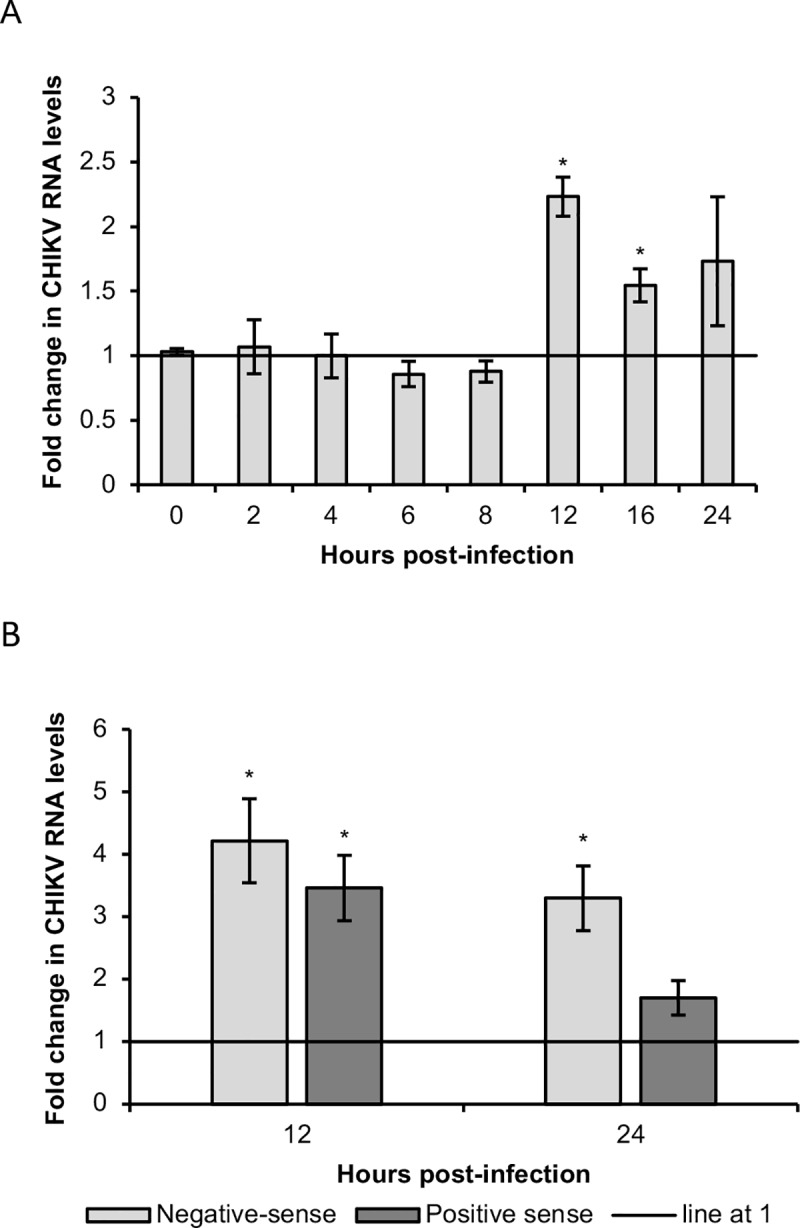
Changes in CHIKV RNA levels upon bortezomib treatment. HeLa cells were infected with CHIKV-122508 and treated with either 0.1 μM bortezomib or vehicle control. Samples were harvested at different time-points post-infection for extraction of total RNA from infected cells. Levels of (A) total CHIKV RNA or (B) strand-specific CHIKV RNA were detected by qRT-PCR. Actin mRNA was used for normalization. The bar graph shows the fold change of CHIKV RNA in bortezomib-treated samples compared to untreated samples. Fold change was calculated using the double delta ct method. Error bars represent standard errors of replicate means from at least two independent experiments. * *P* < 0.05 (Welch’s t-test comparing proteasome inhibitor-treated samples to vehicle control for each time-point).

Given the alterations to CHIKV ns protein levels, we also evaluated the effects of bortezomib on CHIKV RNA levels. Bortezomib treatment increased total CHIKV RNA levels from 12 hpi onwards (Fig 9A), likely due to the increased ns protein levels at 12 hpi (Fig 5). This increasing trend was observed for both positive-sense and negative-sense CHIKV RNA at 12 hpi and 24 hpi (Fig 9B), suggesting that bortezomib induces similar effects on replication of both CHIKV strands. Nonetheless, with no negative effect on CHIKV RNA synthesis, it is clear that the major direct antiviral effect of bortezomib stems from its effect on suppressing structural protein levels, although the precise mechanisms leading to this effect would require more extensive study.

## Discussion

In this study, we explored the inhibitory effects of proteasome inhibitors on CHIKV replication, with some investigation into their mechanism of antiviral action. A range of proteasome inhibitors from different compound classes were found to reduce CHIKV infectious titers in a dose-dependent manner. Bortezomib, which has been FDA-approved for the treatment of multiple myeloma and mantle cell lymphoma, showed dose-dependent reductions of CHIKV titers in several cell lines (BHK21, HeLa and HSMM). Bortezomib-mediated suppression of CHIKV titers was found to occur at an early, post-entry stage of replication. Western blot analysis showed prominent reductions (50 to 80%) in the levels of structural CHIKV proteins (E2, E1 and capsid) in time-course studies as early as 6 hpi, which was confirmed in dose-dependent studies. Unlike the obvious and consistent reductions in structural proteins, nsP2 and nsP3 levels showed a fluctuating trend over time in western blot assays, with elevations observed from 12 to 20 hpi for nsP2 and at 12 hpi for nsP3. Contrastingly, nsP4 levels showed strong elevations across all time-points. The reductions in structural protein levels and elevated nsP4 levels were also recapitulated upon treatment with MG132, MLN2238, lactacystin and ONX-0914, suggesting a common anti-CHIKV mechanism across proteasome inhibitors with different functional groups. Finally, an increase in CHIKV RNA levels was observed from 12 hpi onwards. This increase was observed for both positive- and negative-sense CHIKV RNA. The major direct antiviral effect of bortezomib is likely to result from its effect on suppressing structural protein levels leading to a decrease in progeny virions and infectious titres. More extensive study would be required to identify the precise mechanisms leading to this effect, especially since the ubiquitin-proteasome system is a major pathway for protein degradation in cells and proteasomal inhibition is likely to modify expression levels of a diverse number of cellular proteins and viral host factors, which may affect CHIKV replication in different ways.

In previous studies, the antiviral effects of bortezomib has been attributed to a number of different mechanisms. In cells infected with Rift Valley Fever virus (RVFV), bortezomib reduced the ability of the non-structural S-segment protein (NSs) to form nuclear filaments and decreased its interaction with several host factors by affecting their ubiquitination status. Expression of IFN-β was also increased, suggesting that host antiviral pathways may have further contributed to the inhibition of viral replication [48]. As for severe fever with thrombocytopenia syndrome virus (SFTSV), a phlebovirus related to RVFV, bortezomib treatment reversed the degradation of RIG-I induced by the viral non-structural protein (NS), thereby removing virus-mediated suppression of host innate immunity [49]. Bortezomib has also been found to inhibit replication of dengue virus, likely by suppressing viral egress [50]. While the ubiquitin-proteasome system has been found to be important in the replication of many viruses, the antiviral mechanisms of proteasome inhibition are likely to vary greatly between different viruses [24].

In our study, the increase in nsP4 levels upon bortezomib treatment suggests that the CHIKV nsP4, like that of SFV and SINV, is degraded by the proteasome [30, 31, 42] and the elevated levels are a reflection of its stabilization by bortezomib. Importantly, the elevations in nsP4 levels was not found to be dose-dependent, suggesting that increased nsP4 levels may not be the main factor contributing to the anti-CHIKV effects of bortezomib.

The fluctuating trend observed for nsP2 and nsP3 may be due to multiple competing effects of bortezomib on these proteins. For instance, bortezomib may either increase nsP2/nsP3 expression or reduce their degradation, causing an increase in their levels within infected cells. However, by reducing virion production, bortezomib also restricts viral spread in the monolayer, reducing the overall amounts of protein detected via western blot at other time-points. A similar explanation can be made for the fact that CHIKV RNA levels were observed to be elevated only at 12 hpi, despite increased nsP4 levels from 6 hpi onwards.

While bortezomib caused some increase in ns protein levels, structural protein levels were consistently and significantly decreased at all time-points. This may suggest that bortezomib may affect SG RNA translation differently from G RNA translation. Indeed, translation of G and SG RNA of alphaviruses are known to have different requirements [46]. For instance, the G RNA of SINV requires eukaryotic initiation factors and is believed to be translated in a similar manner as host mRNA, while SINV SG RNA translation proceeds in a non-canonical manner, without the requirement of initiation factors like eIF2 and eIF4, despite bearing a cap structure [46]. These differences in translation requirements for G and SG RNA may explain the different effects of bortezomib on CHIKV ns and structural proteins. A similar phenomenon has been reported for pateamine A, an antiviral compound which was found to block translation of SINV genomic RNA, but not SG RNA [51].

Apart from translational machinery, the stabilization of host and viral factors involved in CHIKV replication may have also contributed to the antiviral effects of bortezomib. For instance, DExH-box RNA helicase 9 (DHX9) has recently been reported to be recruited to CHIKV replication complexes and may function as a switch regulating RNA replication and translation [52]. Importantly, DHX9 was shown to be required for translation of CHIKV ns proteins during the early stages of replication while being degraded by the proteasome during later stages of replication. Another example is RpbI, a catalytic subunit of RNA polymerase II. Recently, Akhrymuk and colleagues reported that the nsP2 of CHIKV, SINV and SFV induce the shut-off of host transcription by causing the proteasomal degradation of RpbI [53]. This process occurs very early in infection (within 6 hpi) and is likely to be crucial in blocking the activation of genes involved in the antiviral response [53]. Prevention of RpbI degradation by proteasomal inhibition may cause an increase in cellular antiviral responses, restricting viral replication. As discussed previously, modulation of antiviral responses has also been reported for bortezomib-mediated inhibition of viruses like RVFV and SFTSV. The alphavirus nsP3 has also shown some involvement in the ubiquitin-proteasome system. Varjak and colleagues (2010) reported that the nsP3 of SFV carries a degradation signal at its C-terminus and is degraded by the proteasome [54]. While this has not been reported for CHIKV nsP3, our data hints at a similar possibility. In dose-dependent studies, treatment with 0.025 μM of bortezomib caused CHIKV nsP3 levels to increase by about 30% above the vehicle control. At higher concentrations of bortezomib, however, nsP3 levels showed a dose-dependent decrease, which may partly be explained by restriction of virus spread in cell monolayers at these concentrations.

The alterations in ns and structural protein levels upon bortezomib treatment also reflect a similar phenotype recently described for a SINV mutant with increased nsP1 capping activity [55]. This mutant over-produced ns proteins and had somewhat elevated levels of viral RNA synthesis. Importantly, it also had slightly reduced synthesis of structural proteins and strongly reduced infectious virion production [55]. These similarities indicate that disrupting the balance between ns and structural protein levels is detrimental for alphavirus replication, perhaps by altering the cellular environment in a way that is unfavorable for virion formation and/or by directly interfering with virion formation. Indeed, nsP2 has been recently identified as component of alphavirus virions [56] and there is genetic evidence that functional linkage of nsP2 and capsid protein is important for alphavirus replication [57, 58]. Therefore, it is plausible that major changes in their ratio, similar to what is observed during bortezomib treatment, would have a negative impact on viral replication.

Another important consideration is that a number of proteasome inhibitors also inhibit other cellular proteases. For instance, bortezomib exhibits off-target activity against some intracellular proteases, including cathepsins A and G, although the IC_50_ (50% inhibitory concentration) for non-proteasomal targets is about 10-fold to 100-fold higher than that required for the inhibition of the proteasome [59]. In addition, lactacystin also inhibits cathepsin A [60]. Results from western blot studies showed that proteasome inhibitors apart from bortezomib caused similar changes to CHIKV proteins, suggesting a common antiviral mechanism across proteasome inhibitors with different functional groups. While it is likely that the antiviral effects of the inhibitors used in this study stem from their action on the proteasome rather than non-proteasomal targets, downstream studies confirming this would be essential.

Given the pleiotropic effects of proteasome inhibitors on host cells, the dissection of antiviral mechanisms requires extensive investigation to determine which pathways are major contributors to their inhibitory effects. Whether the antiviral effects of bortezomib are a result of inhibition of translation of SG RNA, modification of levels of essential host factors, modulation of antiviral pathways, changes to the ratio of ns to structural proteins, or even effects on non-proteasomal targets, it is clear that further evaluation into the potential for these compounds to be used as CHIKV antivirals is warranted.

A limitation of bortezomib is its narrow therapeutic window [61]. In cancer patients, the most significant adverse effect reported is peripheral sensory neuropathy, which is reported in 31% to 55% of patients [62]. It is important to note, however, that bortezomib-induced toxicity is related to accumulated dose, with neuropathy usually surfacing within the first five cycles of treatment, in a treatment plan where each cycle is six weeks long [37, 59, 63, 64]. However, for CHIKV infection, treatment schedules are not expected to last as long, given that the acute phase is resolved within two weeks. Furthermore, altering the route of administration of bortezomib from intravenous to subcutaneous has been found to significantly reduce instances of neuropathy in patients, while retaining similar efficacy [37]. In addition, our study has shown that proteasome inhibitors with various functional groups are able to inhibit CHIKV and cause similar changes to viral proteins. This indicates that *in vivo* evaluation of proteasome inhibitors for treatment of CHIKV should not be limited to bortezomib. Rather, the use of newer generation proteasome inhibitors like MLN2238, which has an improved toxicity profile and is administered orally, should also be explored [65]. Another option is to chemically modify the structure of the proteasome inhibitor such that it is active only upon cleavage by viral proteases in infected cells. Buckley and colleagues (2011) reported the design and synthesis of a proteasome inhibitor with a large chemical moiety that blocks its entry into the proteasome unless it is cleaved by the human immunodeficiency virus (HIV) protease [66]. This steric-capped proteasome inhibitor was able to inhibit human proteasome activity in cell-free assays only when pre-treated with HIV protease. Indeed, a similar method could be employed to limit toxicity of bortezomib and other proteasome inhibitors in the context of CHIKV infection. Extensive optimisations of the modified proteasome inhibitor structure, together with detailed *in vitro* and *in vivo* validation would be needed to confirm efficacy.

## Materials and methods

### Cell lines and virus

Cell lines used in this study include BHK21 baby hamster kidney cells (ATCC CCL-10), HeLa cervical cancer epithelial cells (ATCC CCL-2), human skeletal muscle myoblasts (HSMM, Lonza) and C6/36 from *Aedes albopictus* embryonic tissue. BHK21 cells were cultured in Roswell Park Memorial Institute-1640 media (RPMI-1640, Sigma-Aldrich) supplemented with 10% inactivated fetal calf serum (FCS, Capricorn Scientific). HeLa cells were cultured in Dulbecco’s Modified Eagle’s media (DMEM, Sigma-Aldrich) containing 10% inactivated FCS. HSMM cells were cultured in SkGM (Skeletal Muscle Cell Growth Medium, Lonza) with supplied growth factors and 10% inactivated FCS. Throughout the study, BHK21, HeLa and HSMM cultures were incubated at 37°C in a humidified incubator with 5% CO_2_. C6/36 cells were cultured in Leibovitz’s L-15 medium (Sigma-Aldrich) containing 10% inactivated FCS and incubated at 28°C without humidification.

This study used two strains of CHIKV East/Central/South African (ECSA) genotype: CHIKV-122508 (SGEHICHID122508, Accession No.: FJ445502.2) and CHIKV-0708 (Singapore/07/2008). CHIKV-122508 was obtained from the Environmental Health Institute (EHI), National Environment Agency (NEA), Singapore. CHIKV-0708 was obtained from A/P Raymond Lin (National Public Health Laboratory, Ministry of Health, Singapore). CHIKV-122508 was propagated in C6/36 cells, while CHIKV-0708 was propagated in BHK21 cells.

### Screening of proteasome inhibitors against CHIKV

Proteasome inhibitors were screened in post-treatment assays. BHK21 cells were seeded onto 96-well plates (Corning) at a density of 12,500 cells per well and incubated overnight at 37°C. Cell monolayers were incubated with CHIKV-122508 at a MOI of 10 for 1.5h at 37°C. After this inoculum was removed, cells were washed twice with PBS and incubated with proteasome inhibitors diluted to selected concentrations in RPMI-1640 medium with 2% FCS. After an incubation period of 24h at 37°C, the plates were frozen at -80°C prior to quantification of viral titer in cell culture supernatants via plaque assays. Plaque assays were performed using previously described methods [67].

For bortezomib, a pre-treatment assay was also performed, where cells were treated with bortezomib prior to infection with CHIKV. BHK21 monolayers were incubated with various concentrations of bortezomib for 2h at 37°C before being washed twice with PBS. Treated cells were then infected with CHIKV-122508 at MOI 10 for 1.5h. Infected monolayers were washed twice with PBS before being incubated in RPMI-1640 with 2% FCS (without bortezomib) for 24h at 37°C. Plates were stored at -80°C prior to performing plaque assays for titration.

### Validation of bortezomib activity in HeLa and HSMM

Post-treatment experiments similar to the ones conducted for BHK21 were performed on HeLa and HSMM. HeLa and HSMM cells were seeded in 96-well plates at densities of 15,000 and 20,000 cells per well, respectively, and incubated overnight. HeLa monolayers were infected with different strains of CHIKV at 10 for 1.5h at 37°C and washed twice with PBS. For HSMM, cells were infected with CHIKV-122508 at MOI 10 for 1.5h at 37°C and washed twice with PBS. In this and other experiments, MOI for HeLa and HSMM cells was counted based on virus titer measured in BHK21 cells and actual level of productive infection is expected to differ across cell lines [68]. After the washing step, infected cells were incubated with bortezomib at various concentrations for 48h (HeLa) or 24h (HSMM) at 37°C, prior to being harvested for plaque assays. In order to measure the therapeutic window of bortezomib in CHIKV-infected HeLa cells, the selectivity index (SI) was calculated. It is expressed as a ratio of the CC_50_ (50% cytotoxic concentration) to EC_50_ (50% effective concentration). The EC_50_ of bortezomib in HeLa cells was defined as the concentration of bortezomib required to reduce virus titer by 50%. The CC_50_ was defined as the concentration of bortezomib required to reduce cell viability by 50%.

### Cell viability assay

BHK21, HeLa and HSMM cells were seeded on 96-well plates at densities of 12,500, 15,000 and 20,000 cells per well, respectively. After overnight incubation, cell culture media was replaced with media containing proteasome inhibitors or vehicle control at various concentrations and incubated for 24h (BHK21 and HSMM) or 48h (HeLa) at 37°C. Cell viability was then determined using the alamarBlue® cytotoxicity assay (Invitrogen) as previously described [69].

### Time-of-addition and time-of-removal studies

HeLa cells were plated at a density of 15,000 cells per well in 96-well plates. After overnight incubation, HeLa monolayers were infected with CHIKV-122508 at MOI 10 for 1.5h and washed twice with PBS. For time-of-addition experiments, 0.1 μM bortezomib was added at different time-points after the 1.5h infection period. In time-of-removal experiments, bortezomib was added immediately after the 1.5h infection period and removed at different time-points. Plates from both time-of-addition and time-of-removal experiments were harvested at 48 hpi for titration via plaque assays.

### Studies on CHIKV protein levels

HeLa cells were seeded onto 6-well plates at a density of 750,000 cells per well and incubated overnight. Cell monolayers were then infected with CHIKV-122508 at MOI 10 for 1.5h and washed 3 times with PBS. Infected cells were treated with proteasome inhibitors or vehicle control and harvested at indicated time-points up to 24 hpi.

During harvesting, cells were washed once with PBS and incubated with cell lysis solution at 4°C for 10 min, with rocking. The cell lysis solution used was M-PER (Mammalian Protein Extraction Reagent, ThermoFisher Scientific) with added Halt Protease Inhibitor (ThermoFisher Scientific) and EDTA (ThermoFisher Scientific). Cells were then scraped and centrifuged for 10 min at 10,000 x *g* and 4°C to pellet cell debris. Protein-containing supernatants were stored at -80°C prior to SDS-PAGE and Western blot.

### Western Blot

Samples were thawed on ice and quantitated using the Bio-Rad Protein Assay Dye Reagent Concentrate according to the manufacturer’s instructions. Equal amounts of protein were loaded into each well. Cell lysates were separated by SDS-PAGE and proteins were transferred to nitrocellulose membranes in a semi-dry transfer protocol using the Trans-Blot Turbo Transfer System (Bio-Rad). Blots were then blocked overnight and incubated with primary antibodies for 1h while rocking at room temperature. Dilution of anti-CHIKV nsP2 (1:50), anti-CHIKV nsP3 (1:100), anti-CHIKV E1 (1:200), anti-CHIKV capsid (1:300), anti-eIF2α (1:500), anti-phospho-eIF2α (S51) (1:500) and anti-β-actin (1:10,000) antibodies were performed in 5% BSA in TBST. Anti-CHIKV E2 (1:100) was diluted in 5% skim milk in TBST while anti-CHIKV nsP4 (1:1000) was diluted in 2% BSA in PBST. After primary antibody incubation, blots were washed 4 times with TBST or PBST (for nsP4 blots) for 5 min each before incubation with appropriate secondary antibodies (goat horseradish peroxidase (HRP)-conjugated anti-rabbit or anti-mouse IgG), for 1h. After the incubation period, blots were washed 4 times and developed by the enhanced chemiluminescence (ECL) method using SuperSignal West Dura Extended Duration Substrate (ThermoFisher Scientific). Detection was carried out on a C-DiGit Blot Scanner (LI-COR) and band intensities were quantified on the accompanying LI-COR Image Studio programme.

### Measurement of changes in CHIKV RNA levels upon bortezomib treatment

HeLa cells were seeded on 24-well plates (80,000 cells per well) and incubated overnight before infection with CHIKV-122508 at MOI 10 for 1.5h at 37°C. Cell were then washed 3 times with PBS before being incubated with 0.1 μM bortezomib or vehicle control. Cells were washed once with PBS before total cellular RNA was extracted using the RNeasy Mini Kit (Qiagen) according to the manufacturer’s instructions.

Samples were then assayed in a one-step qRT-PCR protocol using the SYBR Green Quantitative RT-qPCR kit (Sigma-Aldrich). Reactions (25 μl) contained 12.5 μl of 2X SYBR green master-mix, 25 units of M-MLV reverse transcriptase, 0.2 μM each of forward and reverse primers and 50 ng of RNA. Primers targeting the capsid gene in the CHIKV genome were used (forward primer: 5’-GCGGTACCCCAACAGAAG-3’; reverse primer: 5’-GGTTTCTTTTTAGGTGGCTG-3’). Actin was also detected as a housekeeping gene for normalisation across samples (forward primer: 5’-AGCGCGGCTACAGCTTCA-3’; reverse primer: 5’-GGCGACGTAGCACAGCTTCT-3’). Reactions were carried out in an Applied Biosystems StepOnePlus qPCR system, beginning with a 30 min reverse transcription step at 42°C. This was followed by a 5 min step at 95°C for *Taq* polymerase activation. 40 cycles of 95°C for 15 sec and 60°C for 1 min were carried out for amplification and fluorescence measurement. This was followed by melt-curve analysis to verify melting temperatures of PCR products. The fold changes of bortezomib-treated samples compared to untreated samples were calculated by the double-delta ct method.

### Statistical analysis

Welch’s t-test or a one-way analysis of variance (ANOVA) followed by Dunnett’s post-test was carried out to determine whether there were significant differences (*P* < 0.05) between proteasome-treated samples and vehicle controls (GraphPad Prism 8).

## Supporting information

S1 Table List of proteasome inhibitors tested in cell-based inhibition studies. ^a^MLN-9708 is converted to MLN-2238 in vivo.
(DOCX)

S2 Table Predicted ubiquitination sites within CHIKV protein sequences. Ubiquitination sites were predicted using UbPred. Only lysine residues predicted to be ubiquitinated with medium and high confidence levels are shown.
(DOCX)
