# Supplementary material for: Bortezomib inhibits chikungunya virus replication by interfering with viral protein synthesis
Source: PLoS Negl Trop Dis. 2020 May 29;14(5):e0008336. doi: 10.1371/journal.pntd.0008336 (PMC7286522; doi:10.1371/journal.pntd.0008336)
Supplement: S1 Table — aMLN-9708 is converted to MLN-2238 in vivo. (DOCX) [file pntd.0008336.s001.docx]

**S1 Table: List of proteasome inhibitors tested in cell-based inhibition studies.**

^a^MLN-9708 is converted to MLN-2238 in vivo.

| **Class** | **Proteasome Inhibitor** | **Structure** |
| --- | --- | --- |
| **Peptide Aldehydes** | MG-132 |  |
| **Peptide Boronates** | Bortezomib |  |
|  | Delanzomib |  |
|  | MLN-9708^a^ |  |
|  | MLN-2238^a^ |  |
| **Peptide Epoxyketones** | Carfilzomib |  |
|  | Epoxomicin |  |
|  | Oprozomib |  |
|  | ONX-0914 |  |
| **β-Lactones** | Lactacystin |  |
| **Anthracycline Derivatives** | Aclacinomycin A |  |
