# Supplementary material for: Bortezomib inhibits chikungunya virus replication by interfering with viral protein synthesis
Source: PLoS Negl Trop Dis. 2020 May 29;14(5):e0008336. doi: 10.1371/journal.pntd.0008336 (PMC7286522; doi:10.1371/journal.pntd.0008336)
Supplement: S2 Table — Ubiquitination sites were predicted using UbPred. Only lysine residues predicted to be ubiquitinated with medium and high confidence levels are shown. (DOCX) [file pntd.0008336.s002.docx]

**S2 Table: Predicted ubiquitination sites within CHIKV protein sequences.**

Ubiquitination sites were predicted using UbPred. Only lysine residues predicted to be ubiquitinated with medium and high confidence levels are shown.

| **CHIKV Protein** | **Position** | **Score** | **Confidence Level** |
| --- | --- | --- | --- |
| Nsp1 | 495 | 0.79 | Medium |
| Nsp3 | 245 | 0.74 | Medium |
| Nsp4 | 34  39 44 50 51 | 0.85  0.75  0.74  0.85  0.79 | **High**  Medium  Medium  **High**  Medium |
| Capsid | 182  209 | 0.77  0.71 | Medium  Medium |
| E3 | 29 | 0.72 | Medium |
| E2 | 140  149 | 0.71  0.72 | Medium  Medium |
| E1 | 110  211 | 0.86  0.73 | **High**  Medium |
